# Association Between Dietary Inflammatory Index and S-Klotho Plasma Levels in Middle-Aged and Elderly People

**DOI:** 10.3389/fnut.2022.853332

**Published:** 2022-05-10

**Authors:** Teng-Chi Ma, Jing Zhou, Chen-Xi Wang, Min Fang, Feng Gao

**Affiliations:** Affiliated Hospital of Yan'an University, Yan'an, China

**Keywords:** aging, dietary inflammatory index, Klotho, inflammation, diet

## Abstract

**Background and Aims:**

Soluble Klotho (S-Klotho) is a protein that has anti-aging properties. Dietary inflammation index (DII) is closely related to various age-related diseases. However, whether DII is related to S-Klotho plasma levels is still controversial. It was the goal of this study to examine the link between DII and S-Klotho in middle-aged and elderly people.

**Methods:**

Between 2007 and 2016, five NHANES cycles were conducted, with 12,315 middle-aged and elderly (aged 40–79) participants having S-Klotho tests and submitting dietary recall data. The inflammatory potential of a diet was determined using the DII. To determine the plasma levels of S-Klotho, we employed a solid-phase sandwich enzyme-linked immunosorbent assay (ELISA).

**Results:**

There was a negative correlation between DII and S-Klotho plasma levels. In the threshold effect analysis model, the breakpoint was DII=1.3, and the negative correlation was more obvious when DII < 1.3 (β = −10.6, *p* = 0.001). When DII > 1.3, the correlation disappeared (*p* = 0.355). There may be a threshold saturation effect.

**Conclusion:**

In middle-aged and older individuals, there is a negative connection between the pro-inflammatory dietary pattern as evaluated by DII and the plasma level of S-Klotho. Given the rationale for the findings and the study's limitations, the fundamental mechanisms generating inflammation warrant additional exploration.

## Introduction

Klotho, a transmembrane protein, possesses extraordinary anti-aging capabilities (1). Clotho is the name of one of Greek mythology's three fate goddesses, a just but forgiving divinity. As a result, when researchers discovered a protein that “secures the human lifeline,” they gave it the name Klotho. Klotho protein can be found in two different forms: membrane and secretory. The hydrolysis of protein on the Klotho membrane produces the exfoliated form (S-Klotho) of Klotho protein. In comparison to membrane Klotho protein, S-Klotho protein is more prevalent in the human body, with S-Klotho found in urine, blood, and cerebrospinal fluid (CSF) (2–4). As a humoral factor, it performs a variety of biological functions in the circulatory system, including inflammation regulation, antioxidation, and senescence prevention (5, 6). By contrast, a shortage of Klotho can result in a variety of age-related diseases, including atherosclerosis, endothelial dysfunction, decreased bone mineral density, osteoporosis, skin atrophy, and cognitive impairment (7–11). As the population is getting old, the number of the elderly population is growing, age-related diseases are also on the rise, and the disability burden of age-related diseases is expected to increase. Therefore, it is of great significance to understand the factors related to anti-aging.

Dietary patterns largely determine life expectancy (12). There is a great deal of evidence that many foods, nutrients, and non-nutritious food ingredients can regulate inflammation in both acute and chronic ways (13). Considering the anti-inflammatory and pro-inflammatory regulatory potential of nutrients, a lot of interest has been aroused by the inflammatory burden of diet. The pro-inflammatory diet pattern is related to some age-related systemic diseases, including malignant tumors, cardiovascular metabolic diseases, diabetes, and so on (14–16). Furthermore, given the frequency of food intake, the dynamic equilibrium of chronic inflammation is more dependent on diet than on medicine use. Then, by properly managing dietary components associated with inflammation, several disorders caused by inflammatory pathways can be avoided or cured. Rather than a single assessment based on nutrition, the inflammatory load of food is investigated more thoroughly in certain ways. From this point of view, a method of describing and measuring the inflammatory potential of a person's diet can help develop tailored and accurate dietary intervention and health maintenance strategies. Using inflammatory biomarkers, the dietary inflammation index (DII) has been validated in several groups to help determine the inflammogenic potential of certain people's diets (17). Therefore, high DII (pro-inflammatory diet) may be related to a grown risk of chronic disease or all-cause death (18, 19).

Aging is an inevitable process throughout life. However, dietary patterns reflect years to decades of inflammation in the body, which can be inferred chronic inflammation may be closely related to aging. DII has been adopted for studies to better know the relationship between DII and disease. However, as far as we know, the relationship between the Mediterranean diet and S-Klotho has been studied, and there is only one article on DII and S-Klotho in middle-aged people, and the sample size is small (*n* = 73) (2), so it seems to be very important to find out whether the pro-inflammatory diet can regulate the plasma level of S-Klotho in humans. The goal of this study is to explore the connection between DII and S-Klotho in middle-aged and elderly adults using data from the National Health and Nutrition Examination Survey (NHANES 2007–2016).

## Materials and Approaches

### Participants

Because S-Klotho was evaluated exclusively within that period, the research design comprised data from five National Health and Nutrition Examination Survey cycles (NHANES). The National Center for Health Statistics undertakes a hierarchical, multi-phase assessment of the non-institutionalized civilian population in the United States of America and the District of Columbia (NCHS). The NHANES is used to assess individuals' health and nutritional status and to monitor changes over time. Data collection methods included interviews, physical examinations, and laboratory testing, and all NHANES surveys conducted between 2007 and 2016 were examined and approved by the Centers for Disease Control and Prevention (CDC) and the National Center for Health Statistical Research (NCHS) Ethics Review Committees; All participants provided written informed consent. For this research, data on NHANES2007-2008, 2009-2010, 2011-2012, 2013-2014, and 2015-2016 were searched. The inclusion criteria were as follows: original serum samples of participants aged 40–79 were tested for S-Klotho and answered dietary interviews on total nutrient intake. Excluding people with unreliable dietary records, the total sample size was 12,315. We follow the expansion of enhanced observational research in Nutrition Epidemiology (STROBE-NUT) (Supplementary Table S1) (20).

### Dietary Inflammatory Index (DII)

The DII is a literature-based technique for continuously classifying individual diets from the most anti-inflammatory to the most pro-inflammatory. The DII's development and validation have been detailed elsewhere. DII is a scoring approach that anticipates the mean and standard deviation (SD) for each coefficient using dietary intake data from regionally relevant world datasets. This study calculated the DII scores for 26 commonly consumed foods: Energy (Kcal), Protein (Gm), Carbohydrates (Gm), Total Sugar (Gm), Dietary Fiber (Gm), Total Fat (Gm), Total Saturated Fatty Acids (Gm), Total Monounsaturated Fatty Acids (Gm), Total Polyunsaturated Fatty Acids (Gm), Cholesterol (Mg), Vitamin E, -tocopherol (Mg), Flavane-3-ol, flavone, flavonol, and green/black tea. First, we calculated every food coefficient and every participant's Z-score. Next, every individual Z-score is transformed into a central percentile. Thirdly, a standardized global inflammatory effect score was adopted to multiply every central percentile. Each participant's DII score is, then, summed up. We determined the highest quartile of DII scores (representing a pro-inflammatory diet) and the lowest quartile of DII scores (showing an anti-inflammatory diet) based on **Table 3** (17).

### S-Klotho Plasma Levels

During the five cycles of the NHANES project, blood samples were taken from the original serum samples that were available to participants aged 40–79. The samples were stored on dry ice and each package was examined by personnel in the receiving area of the laboratory. The sample is scanned, the data is compared with the data on the received electronic manifest, and input into the laboratory information system. All samples are stored at −80°C until predetermined batches of samples are provided to technicians for analysis every day. S-Klotho is on basis of a solid-phase sandwich enzyme-linked immunosorbent assay kit (IBL International, Japan). The analysis results are automatically transmitted from the instrument to the laboratory Oracle management system, and the regional supervisor evaluates the results. Samples with repetitive results of more than 10% are marked as repetitive analysis. If the value of the quality control sample is not within the 2SD range of the specified value, the entire analysis run will be rejected and the sample analysis will be repeated.

### Assessment of Covariates

The calculation of body mass index (BMI) was made on basis of height and weight (kg/m^2^). At the end of a normal exhalation, the waistline at the midpoint between the ilium and the base of the ribs was measured. Following a 5-min sit-down period and establishing the maximum inflation level (MIL).

#### Blood Pressure

Blood pressure (BP) was manually measured in all eligible patients using a Baumanometer-calibrated mercury real gravity wall sphygmomanometer (W. A. Baum). The circumference of the upper arm is used to calculate the size of the cuff. All examiners have gone through a standardized training program. After sitting silently for 5 min in the mobile testing center, the participants were measured three times in a row. Unless unique participant conditions prevented it, measurements were taken with the right arm, except when the left arm was used. The measurement interval is 30 seconds.

#### Use of Medication

We used the NHANES variable “RXDUSE” to determine the medication status. During the interview, participants were asked, “have you used or taken any prescription drugs in the past month?” Then, participants who reported the use of prescription drugs were asked to show the container of prescription drugs, so that the interviewer could record the relevant information. The analysis did not include dietary supplements reported for this concern.

### Statistical Analysis

Data was collected from nhanesR (http://ckr123.synology.me:3838/nhanesR/) on the NHANES project for five consecutive complete cycles from 2007 to 2016. The statistical packages R (The R Foundation; http://www.r-project.org; version 3.5.3) and EmpowerStats (www.empowerstats.com; X&Y Solutions Inc.) were adopted to analyze data. All analyses are calculated using sample weights according to analysis guidelines edited by the National Institutes of Health because NHANES aims at producing data that represent the non-institutionalized civilian population of the United States. The weighted chi-square test was carried out for the classified variables, and the *P*-value of the continuous variables was calculated with the weighted linear regression model. A simple linear regression model was set up to test the correlation between DII and S-Klotho plasma levels: model 1, no elements have been adjusted; model 2, modified for age, sex, and race; and model 3, the adjustment of all covariates in Table 1 was made for further subgroup analysis. The additive model and smooth curve fitting were generalized to explore the potential non-linear correlation. Furthermore, the inflection point is calculated by using a two-stage linear regression model. *P* < 0.05 was of statistical significance.

**Table 1:** Baseline characteristics of participants (*N* =12,315).

**Characteristic**	**Klotho levels quartiles, pg/mL**				***P*** **-value**
	**Q1**	**Q2**	**Q3**	**Q4**	
	** <654.7**	**≥654.7 to <802.5**	**≥802.5 to <993.3**	**≥993.3**	
No. of participants	3,079	3,077	3,079	3,080	
Age(years)	59.05 (11.11)	58.00 (10.85)	57.33 (10.70)	56.51 (10.60)	<0.001
Sex (%)					<0.001
Men	49.06	51.00	48.07	42.52	
Woman	50.94	49.00	51.93	57.48	
Race/ethnicity (%)					<0.001
Mexican American	6.61	6.22	6.97	6.98	
Other Hispanic	3.96	4.48	4.83	5.53	
Non-Hispanic white	73.83	75.75	73.42	68.12	
Non-Hispanic black	9.32	7.26	7.67	12.77	
Other race/ethnicity	6.27	6.29	7.11	6.60	
Energy intake (kcal/day)	1927.49 (882.96)	1936.99 (898.62)	1935.79 (893.63)	1916.92 (880.57)	0.800
DII	1.04 (1.75)	0.91 (1.80)	0.91 (1.80)	0.92 (1.83)	0.001
Body mass index (kg/m^2^)	29.99 (6.36)	29.82 (6.64)	29.72 (6.74)	29.64 (6.92)	0.196
Waist circumference (cm)	103.10 (14.67)	102.29 (15.18)	101.46 (15.21)	100.70 (15.78)	<0.001
Use of medication %	72.26	69.00	68.14	67.20	<0.001
SBP(mmHg)	128.80 (18.36)	128.10 (17.96)	126.83 (18.09)	127.17 (17.95)	<0.001
DBP(mmHg)	71.14 (12.48)	71.88 (12.25)	72.29 (11.63)	72.12 (12.22)	0.001

*Mean (±SD) for continuous variables: the P-value was calculated by the weighted linear regression*.

*Percent for categorical variables: P-value was calculated by weighted chi-square test*.

*DII, dietary inflammatory index; SBP, Syst olic Blood, Pressure; DBP, Diastolic Blood pressure*.

## Results

Table 1 displays the baseline features of the research participants. A total of 12,315 participants participated in the current study. According to the S-Klotho quartile, the characteristics of the target population are shown in Table 1. Overall, there were significant differences in age, sex, race, waist circumference, drug use, and blood pressure distribution among S-Klotho quartiles. Participants in the highest quartile were more likely to be middle-aged, female, had lower DII scores, lower waistline, less drug use, and lower systolic and diastolic blood pressure levels than participants in the lowest quartile of S-Klotho. In terms of energy intake and BMI, no significant difference was observed between the quartiles of S-Klotho.

Based on this univariate analysis, we found significant differences in sex, waist circumference, use of medication, systolic and diastolic blood pressure as a potential confounders (Table 2).

**Table 2:** Univariate analysis for S-Klotho.

	**S-Klotho, pg/mL**	
	**β (95%CI)**	***P*** **-value**
DII	−2.64 (−5.52, 0.24)	0.0721
Energy intake	−0.00 (−0.01, 0.00)	0.1335
Sex	−36.32 (−46.73, −25.91)	<0.0001
Body mass index	−1.30 (−2.09, −0.51)	0.0013
Waist circumference	−1.24 (−1.58, −0.90)	<0.0001
Systolic blood pressure	−0.83 (−1.13, −0.52)	<0.0001
Diastolic blood pressure	0.57 (0.12, 1.02)	0.0133

*Non–standardized β coefficient [95% confidence interval], p-values are provided. DII, dietary inflammatory index*.

We discovered an inverse correlation between DII and S-Klotho plasma levels: (β = −6.1, *P* = 0.001; Table 3).

**Table 3:** Relationship between Dietary Inflammatory Index (DII) and S-Klotho pg/mL.

**Outcome**	**Crude Model**		**Model I**		**Model II**	
	**β (95%CI)**	***P*** **-value**	**β (95%CI)**	***P*** **-value**	**β (95%CI)**	***P*** **-value**
DII	−2.6 (−5.5, 0.2)	0.072	−5.5 (−8.4, −2.6)	<0.001	−6.1 (−9.4, −2.7)	0.001
DII(quartile)
Q1 < −2.36	Reference		Reference		Reference	
Q2 ≥−2.36 to <0.23	−35.6 (−63.2, −8.1)	0.011	−39.4 (−66.8, −12.1)	0.004	−37.4 (−64.9, −9.8)	0.007
Q3 ≥ 0.23 to <1.90	−43.1 (−70.6, −15.9)	0.001	−54.4 (−81.6, −27.2)	0.001	−50.1 (−77.9, −22.1)	0.001
Q4 ≥ 1.90	−34.5 (−61.7, −7.3)	0.013	−50.5 (−77.7, −23.3)	0.001	−46.5 (−75.4, −17.6)	0.001
P for trend	0.251		0.003		0.004	
Stratified by gender
Men	−6.4 (−10.3, −2.5)	0.001	−6.4 (−10.4, −2.5)	0.001	−6.2 (−10.2, −2.3)	0.002
Woman	−3.2 (−7.5, 1.1)	0.147	−4.6 (−8.9, −0.3)	0.035	−2.6 (−6.9, 1.7)	0.240
Stratified by race						
Mexican American	−0.6 (−8.8, 7.5)	0.879	0.2 (−8.2, 8.7)	0.955	0.8 (−7.6, 9.3)	0.845
Other Hispanic	4.7 (−4.5, 13.9)	0.319	2.6 (−6.8, 12.0)	0.591	3.4 (−6.0, 12.9)	0.480
Non-Hispanic white	−4.9 (−8.9, −0.8)	0.019	−6.4 (−10.5, −2.2)	0.002	−5.0 (−9.2, −0.9)	0.018
Non-Hispanic black	2.3 (−6.3, 10.9)	0.600	−3.1 (−11.7, 5.5)	0.479	−1.4 (−10.0, 7.2)	0.750
Other race/ethnicity	−8.2 (−18.0, 1.6)	0.103	−9.6 (−19.4, 0.2)	0.054	−8.7 (−18.5, 1.2)	0.085

*Crude Model: There are no covariates were adjusted*.

*Model I: Age, sex, and race/ethnicity were adjusted*.

*Model II: Age, sex, and race/ethnicity, energy intake, BMI, waist circumference, use of medication, systolic blood pressure, and diastolic blood pressure were adjusted*.

**Figure 1:**
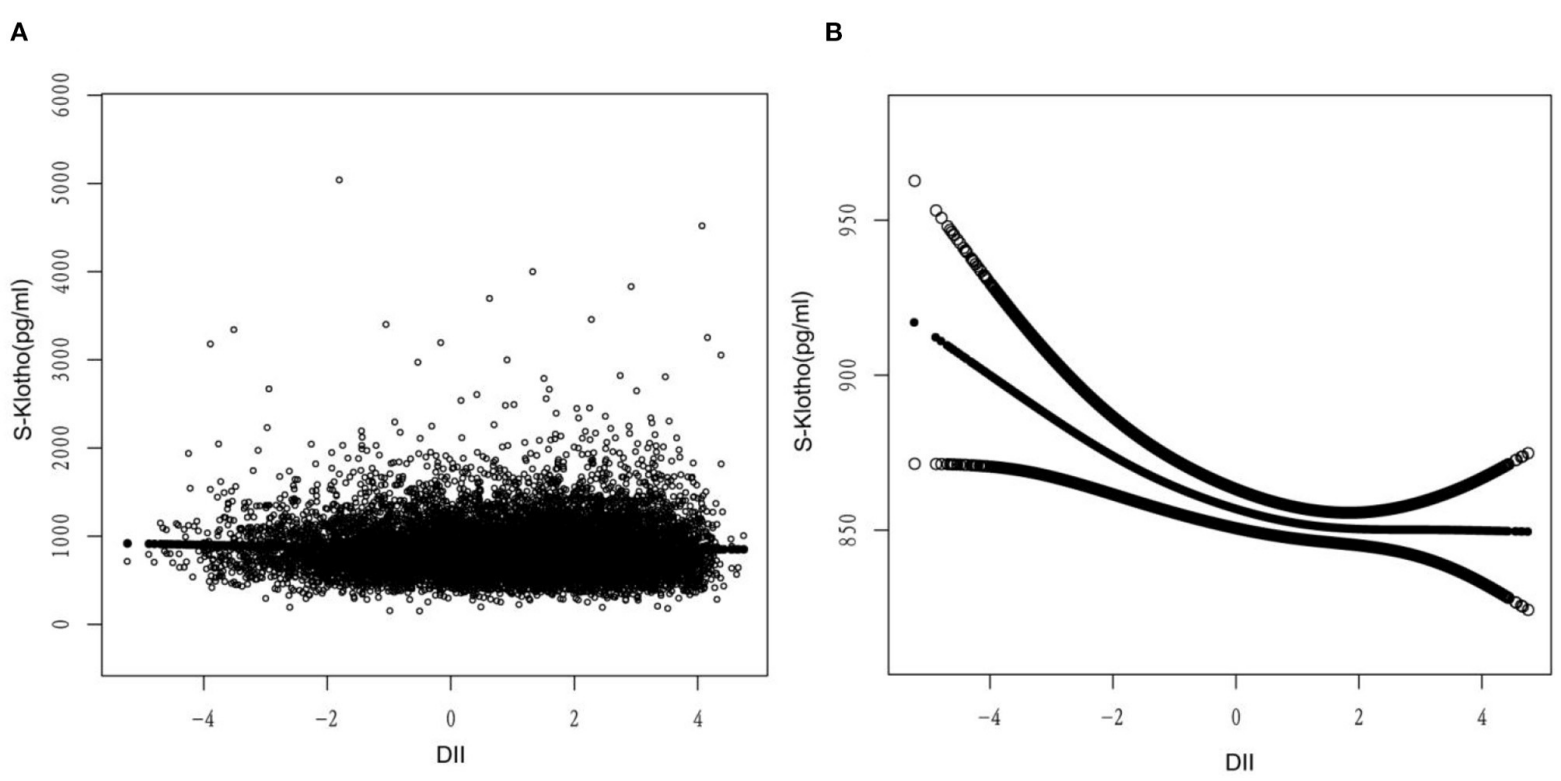
Relationship between DII and S-Klotho. **(A)** Each black dot represents A sample. **(B)** Solid lines represent smooth curve fitting between variables. The black dotted line shows a 95% confidence interval (CI) for the fit. They adjusted for energy intake, age, sex, race, body mass index (BMI), use of medication, and systolic and diastolic blood pressure.

In addition, we handled this relationship using weighted generalized weighted models and smoothing curve fitting (Figure 1). Based on this relationship, we performed an additional threshold effect analysis and found a better fit relative to the linear model to explain the relationship using a non-linear model (log-likelihood ratio = 0.011, Table 4), When the DII score increased by 1 unit, the plasma level of S-Klotho decreased by-10.6 pg/ml (DII < -1.3). This result is consistent with the previous curve fitting plots. Suggesting a possible threshold saturation effect.

**Table 4:** Threshold effect analysis of DII on S-Klotho using the two-piecewise linear regression model.

	**Adjusted β(95% CI)**	***P*** **-value**
Fitting by the standard linear model	−6.1 (−9.4, −2.7)	0.001
Fitting by the two-piecewise linear model		
Inflection point	1.3	
DII <1.3	−10.6 (−15.4, −5.8)	<0.001
DII > 1.3	4.0 (−4.5, 12.4)	0.355
Log-likelihood ratio	0.011	

*Age, sex, and race/ethnicity, energy intake, BMI, waist circumference, use of medication, systolic blood pressure, and diastolic blood pressure were adjusted*.

## Discussion

The current study first establishes a negative connection between DII and S-Klotho plasma levels in middle-aged and elderly subjects in this nationally representative cross-sectional investigation. However, recent research on the DII-S-Klotho connection has been inconclusive. While some feel that eating a higher-quality diet or adopting healthier eating habits will help prevent aging, others remain unsure. One possible explanation for this discrepancy is that these studies used different methods for assessing dietary quality, and dietary quality ratings are dependent on sample data. The majority of research on the link between diet and aging has been on the Mediterranean diet. Studies in the United States (21), Spain (22), and Italy (23) have shown that higher Mediterranean diet scores are associated with anti-aging. In Australia, however, no correlation between the Mediterranean diet and aging was observed (24). Because the Mediterranean diet is not a realistic option for the majority of American adults due to variations in dietary culture and practicality, we employed the DII, an index based on the representative range of dietary intake ingested by humans. This is achieved through the construction of a comprehensive database, including the NHANES database, so it is well-represented nationwide. Only one study in Spain has utilized the DII technique to investigate the association between DII and S-Klotho plasma levels. Interestingly, Lucas et al.'s results (2), based on a survey sample of sedentary middle-aged adults, were just the opposite of ours. Our results support a negative correlation between higher DII scores and higher S-Klotho levels in middle-aged and elderly Americans, which is reasonable and consistent with the hypothetical effect of DII.

Klotho has been linked to aging and is thought to regulate oxidative stress and antioxidant enzymes. Overexpression of S-Klotho can inhibit the expression of retinoic acid-induced gene-I, the activation of NF-κB, and the secretion of proinflammatory cytokines. By contrast, excessive S-Klotho depletion increased the production of pro-inflammatory cytokines, such as tumor necrosis factor-α and IL-1-β, while decreasing the production of anti-inflammatory cytokines, such as IL-10, IL-2, and IL-3 (25). Indeed, DII is a tool that reflects the amounts of six inflammatory markers: IL-1-β, IL-4, IL-6, IL-10, TNF-α, and CRP. The underlying molecular mechanism is that pro-inflammatory substances elevate circulating interleukin (IL) levels (particularly IL-6, IL-1-β, or IL−8) (26–28). As a result, c-reactive protein is extensively generated and released into the circulation by hepatocytes, resulting in more severe systemic inflammation (28). Inflammatory food intake has been shown in the past to have a considerable effect on S-Klotho plasma levels. From a systemic perspective, a pro-inflammatory diet contributes to the elevation of systemic inflammation. Thus, there appear to be grounds to infer that chronic inflammation induced by a pro-inflammatory diet pattern can decrease plasma S-Klotho levels, thereby regulating diet-induced inflammation.

We observed that when the DII score increased by 1 unit, the plasma levels of S-Klotho decreased by −10.6 pg/ml (DII <1.3). These changes in plasma levels of S-Klotho have important clinical significance. In previous studies, when patients with cardiovascular disease were compared with healthy individuals, there were differences in S-Klotho plasma levels in 45 pg/ml (626 and 671 pg/ml, respectively) (29). Furthermore, participants with a blood sugar level of <575 pg/ml had a higher chance of dying from any cause than those with a blood sugar level of more than 763 pg/ml (30). As a result, our results have clinical implications, because variations in DII can affect not only the risk of cardiovascular disease and all-cause mortality but also the rate of aging (29, 30).

The current research has some limitations. First of all, it has a horizontal design that excludes the construction of causality. Second, we don't know whether these outcomes can be extended to young people. More research is needed to see if these benefits may be seen in different populations. Third, the difficulties of correct dietary evaluation, which may be overstated or misclassified, must be considered. Finally, because the level of Klotho protein in many tissues is unknown and can only be tested by biopsy, the results of Klotho protein cannot be predicted.

## Conclusion

In middle-aged and older individuals, there is a negative connection between the pro-inflammatory dietary pattern as evaluated by DII and the plasma level of S-Klotho. Given the rationale for the findings and the study's limitations, the fundamental mechanisms generating inflammation warrant additional exploration.

## Data Availability Statement

The original contributions presented in the study are included in the article/Supplementary Materials, further inquiries can be directed to the corresponding author/s.

## Ethics Statement

The studies involving human participants were reviewed and approved by National Center for Health Statistics Research Ethics Review Board (ERB) for NHANES 2011–2016 (Protocol #2011-17) and NHANES 2007–2010 (Protocol #2005-06) on which data for this analysis was used. Additional details are available at: https://www.cdc.gov/nchs/nhanes/irba98.htm. The patients/participants provided their written informed consent to participate in this study.

## Author Contributions

The study was created by T-CM, who also examined the data. FG revised and edited the text for key intellectual content. All authors evaluated and approved the final manuscript.

## Conflict of Interest

The authors declare that the research was conducted in the absence of any commercial or financial relationships that could be construed as a potential conflict of interest.

## Publisher's Note

All claims expressed in this article are solely those of the authors and do not necessarily represent those of their affiliated organizations, or those of the publisher, the editors and the reviewers. Any product that may be evaluated in this article, or claim that may be made by its manufacturer, is not guaranteed or endorsed by the publisher.
