# Supplementary figures and images for: Association Between Dietary Inflammatory Index and S-Klotho Plasma Levels in Middle-Aged and Elderly People
Source: Front Nutr. 2022 May 10;9:853332. doi: 10.3389/fnut.2022.853332 (PMC9127966; doi:10.3389/fnut.2022.853332)

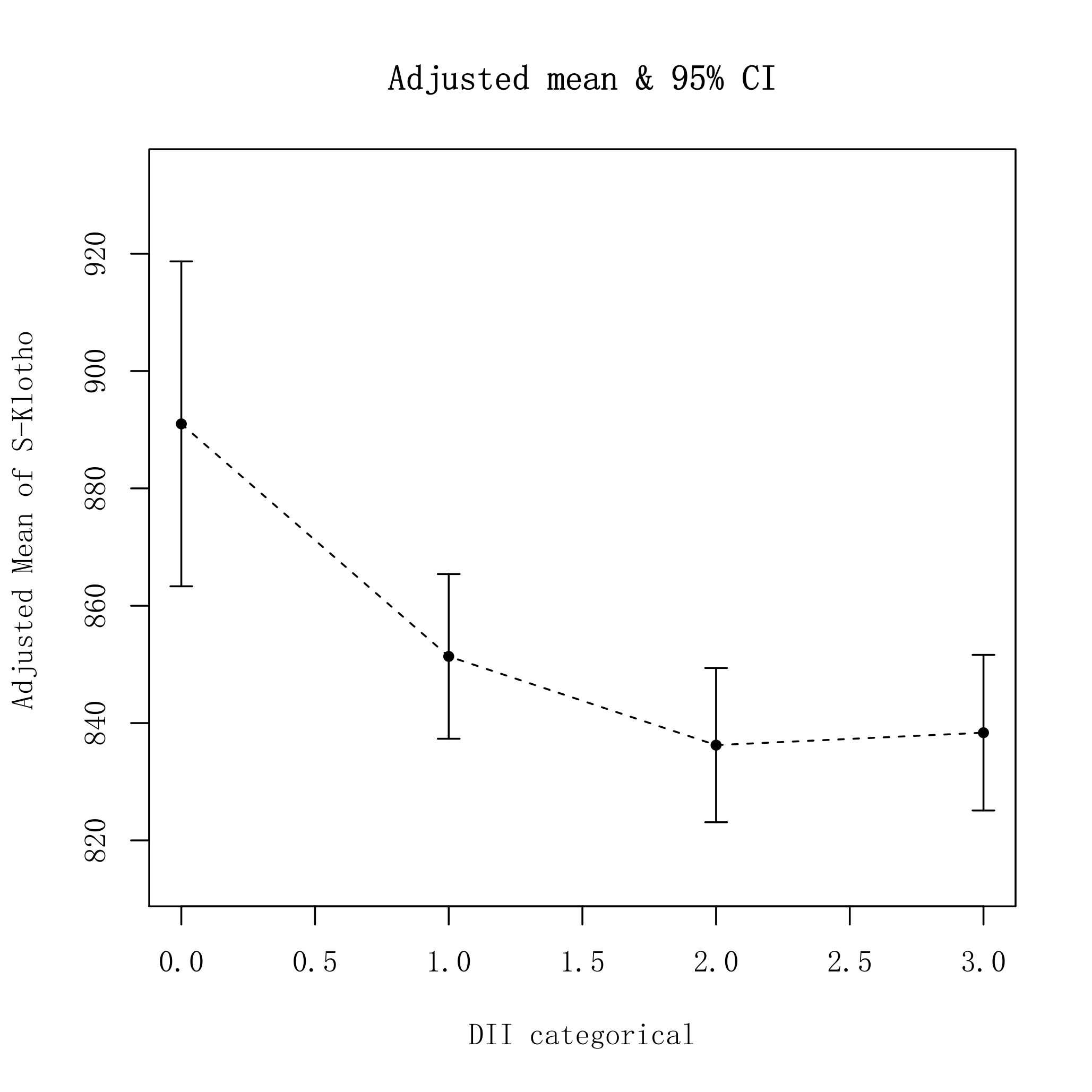

Supplement: Supplementary file 2 [file Image_1.PNG]

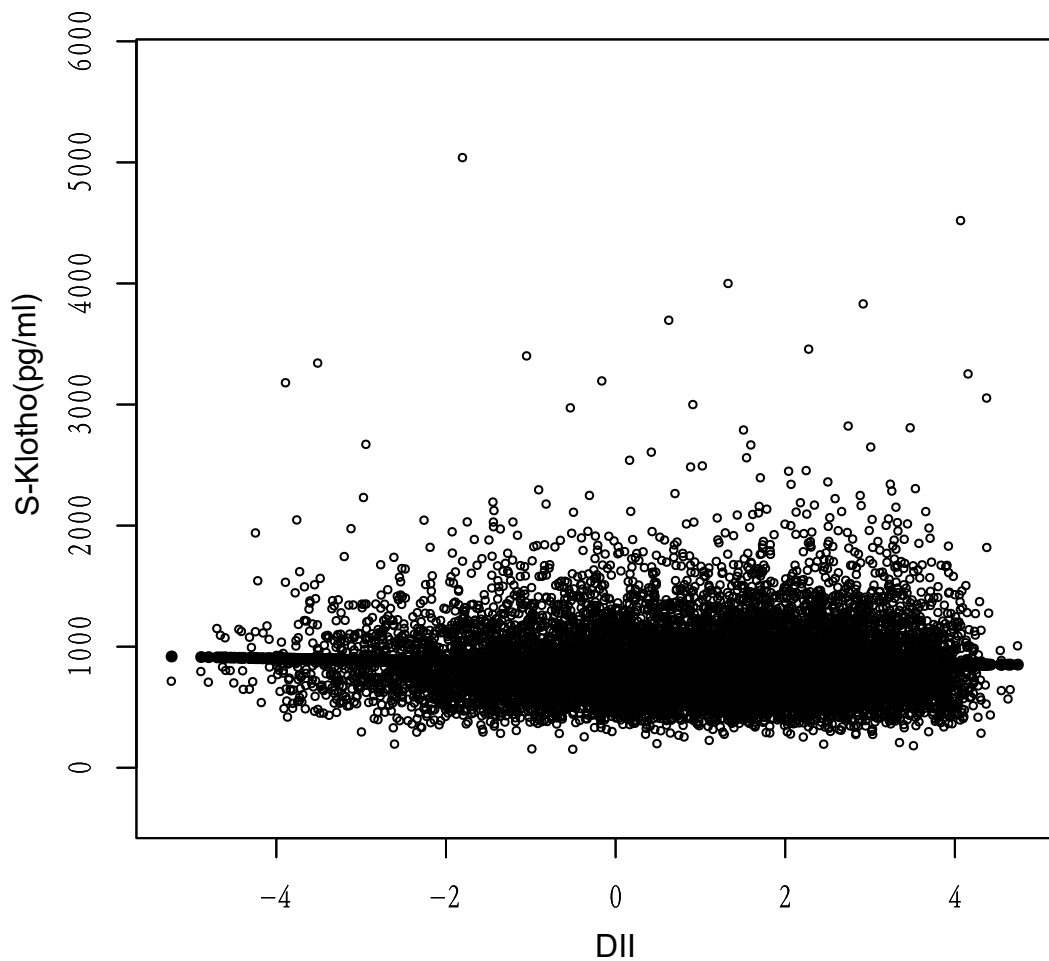

Supplement: Supplementary file 3 [file Image_2.pdf]

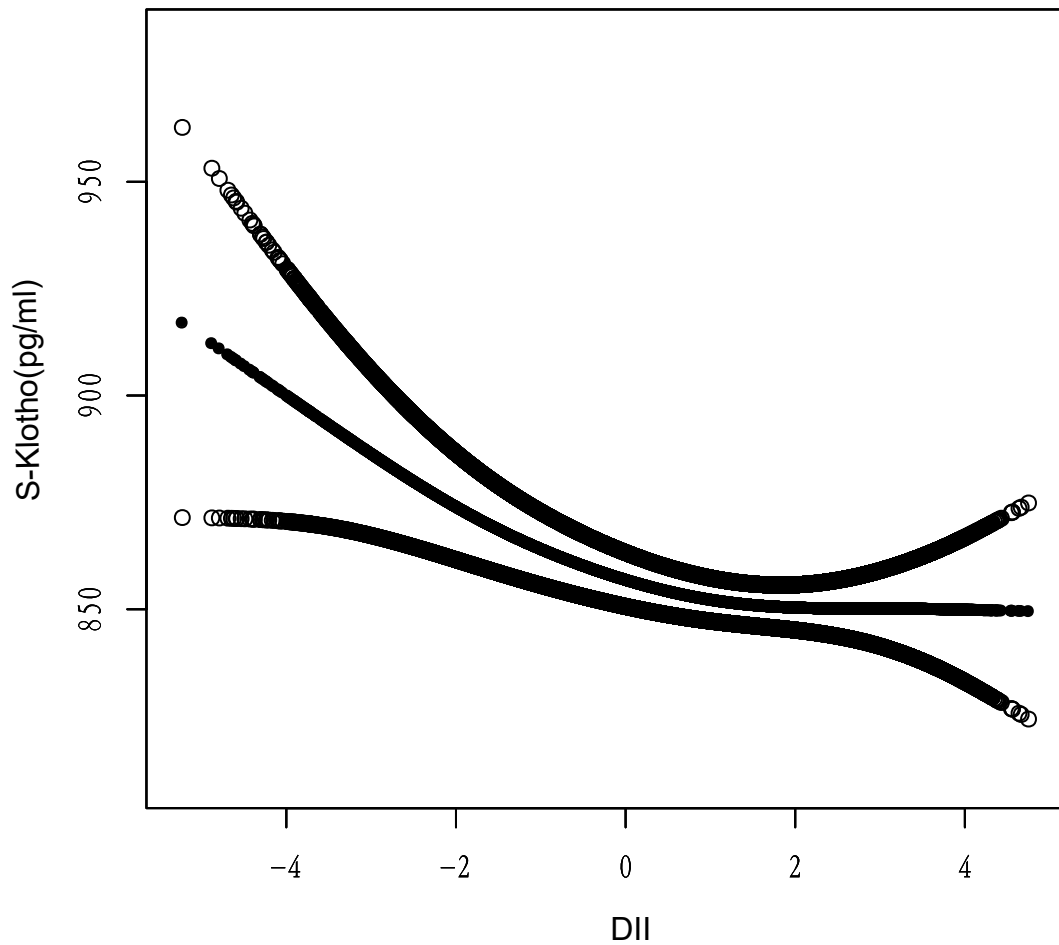

Supplement: Supplementary file 4 [file Image_3.pdf]
